# Spatial Regression Models to Improve P2P Credit Risk Management

**DOI:** 10.3389/frai.2019.00006

**Published:** 2019-05-16

**Authors:** Arianna Agosto, Paolo Giudici, Tom Leach

**Affiliations:** Department of Economics and Management, University of Pavia, Pavia, Italy

**Keywords:** credit risk, systemic risk, contagion, spatial autoregressive models, binary data

## Abstract

Calabrese et al. (2017) have shown how binary spatial regression models can be exploited to measure contagion effects in credit risk arising from bank failures. To illustrate their methodology, the authors have employed the Bank for International Settlements' data on flows between country banking systems. Here we apply a binary spatial regression model to measure contagion effects arising from corporate failures. To derive interconnectedness measures, we use the World Input-Output Trade (WIOT) statistics between economic sectors. Our application is based on a sample of 1,185 Italian companies. We provide evidence of high levels of contagion risk, which increases the individual credit risk of each company.

## 1. Introduction

In recent years, the emergence of financial technologies (fintechs) is redefining the roles of financial intermediaries and introducing many opportunities for consumers and investors. In particular, peer-to-peer (P2P) online lending platforms allow private individuals to directly make small and unsecured loans to private borrowers.

P2P lending business models vary in scope and structure: a comprehensive review is provided by Claessens et al. (2018). Here we specifically refer to the platforms that lend to small and medium enterprises (SME).

While both classic banks and P2P platforms rely on credit scoring models for the purpose of estimating the credit risk of their loans, the incentive for model accuracy may differ significantly.

In a bank, credit risk assessment is conducted by the financial institution itself which, being the actual entity that assumes the risk, is interested to have the most accurate possible model. In a P2P lending platform, credit risk is determined by the platform but the risk is fully borne by the lender. In other words, P2P platforms allow for direct matching between borrowers and lenders.

A factor that penalizes the accuracy of P2P credit scoring models is that they often do not have access to borrowers' data usually employed by banks, such as account transaction data, financial data and credit bureau data. For these reasons, the accuracy of credit risk estimates provided by P2P lenders may be poor. However, P2P platforms involve their users and, in particular, the borrowers, in a continuous networking activity. Data from such activity can be leveraged not only for commercial purposes, as it is customarily done, but also to improve credit risk accuracy.

We believe that networking information can offset the lack of financial and credit behavioral data and improve credit risk measurement accuracy of P2P lenders, but also of banks. There are indeed cases in which also traditional financial intermediaries face lack of information about the borrower. Consider, for example, credit granting to new customers, for whom internal behavioral data—known to be the most predictive in rating models—are not available.

When financial networks are backed by statistical models, inferential statements can be obtained. Important contributions in this framework are Billio et al. (2012); Diebold and Yilmaz (2014); Hautsch et al. (2015); Ahelegbey et al. (2016); Giudici and Spelta (2016), and Giudici and Parisi (2018), who propose measures of connectedness based on similarities, Granger-causality tests, variance decompositions and partial correlations between market price variables.

We improve these contributions, extending them to the P2P context and linking network models, that are often merely descriptive, with econometric models, thus providing a predictive framework.

More specifically, we suggest to use spatial econometrics to study the interconnectedness in the corporate sector. Spatial econometrics incorporates dependence among observations that are in any kind of proximity, not only geographical.

In particular, the model we apply is a logit Spatial Autoregressive model based on an exogenously defined network. The main advantage of this approach over the traditional network analysis is that it can be used as both an early warning model, to forecast the failure of a given company, and as a stress testing technique taking systemic effects into account.

The paper is organized as follows. Section 2 explains the econometric methodology. Section 3 presents the results obtained by applying the proposed methodology to data collected from a European P2P lending information provider. Section 4 concludes.

## 2. Methodology

### 2.1. Spatial Logit

The model we use in this paper has a binary spatial autoregressive structure, whereby the dependent variable is binary and a spatial autoregressive structure is assumed in the underlying latent variable. Taking the latent underlying quantity to be represented by a continuous variable $y_{i}^{*}$, we consider the observation mechanism as

(1) $$\begin{array}{l} y_{i}=\{\begin{array}{cc} 1, & y_{i}^{*}>0\\ 0, & \text{otherwise}, \end{array} \end{array}$$

with *i* = 1, 2, …, *n*.

We implement the spatial structure with an autoregressive model specification, such that

(2) $$\begin{array}{l} Y^{*}=\rho WY^{*}+X\beta +\epsilon , \end{array}$$

where Y^*^ is a continuous random vector, *X* represents an *n* × *k* matrix of explanatory variables with related coefficient vector β, ϵ is the error term and *W* is the spatial lag weight matrix with ρ the associated coefficient, which in our application to defaults will be interpreted as a contagion parameter.

The model implies heteroskedastic errors *e* as follows:

(3) $$\begin{array}{l} Y^{*}={\left(I-\rho W\right)}^{-1}\left(X\beta +\epsilon \right)={\left(I-\rho W\right)}^{-1}X\beta +e, \end{array}$$

where

(4) $$\begin{array}{l} e={\left(I-\rho W\right)}^{-1}\epsilon \end{array}$$

and

(5) $$\begin{array}{l} var\left(e\right)=var\left[{\left(I-\rho W\right)}^{-1}\epsilon \right]=\sigma_{\epsilon }^{2}{\left[{\left(I-\rho W\right)}^{\prime }\left(I-\rho W\right)\right]}^{-1}. \end{array}$$

The defined model has been used by Calabrese et al. (2017) to study default interdependence in the European banking sector. Relative to the estimation, Calabrese and Elkink (2014) have provided a review of the main methodologies for model (3) in the literature. Among the various approaches, we focus on the Generalized Method of Moments (GMM) proposed by Pinkse and Slade (1998). They derive the Generalized Method of Moments (GMM) moment equations from the likelihood function of a spatial error probit model, for which Klier and McMillen (2008) provide the extension to logit models. The GMM approach does not rely on a potentially inaccurate assumption of normally distributed errors and is therefore more robust than maximum likelihood methods.

In general, a GMM estimator is defined by:

(6) θ^≡arg min Θmn(θ)Ωnmn(θ)′,

where *m*_*n*_(θ) are the moment conditions and Ω_*n*_ is a weighting matrix to be determined.

In our case, we have:

$$\begin{array}{l} \theta =\left[\rho ,\beta \right] \end{array}$$

To construct the moments, following Pinkse and Slade (1998) we use the generalized residuals

(7) $$\begin{array}{l} u_{i}=y_{i}-p_{i}, \end{array}$$

where:

$$\begin{array}{l} p_{i}=Pr\left[y_{i}=1\right]=\frac{\exp^{{\left(I-\hat{\rho }W\right)}^{-1}X\hat{\beta }}}{1+\exp^{{\left(I-\hat{\rho }W\right)}^{-1}X\hat{\beta }}} \end{array}$$

It follows from specification (3) that the elements of the spatially lagged dependent vector WY^*^ are correlated with those of the error vector, hence the need for instrumental variables. Following Kelijian and Prucha (1998), who suggest to choose the instruments as a subset of the linearly independent columns of:

$$\begin{array}{l} H=\left\{X,WX,W^{2}X,W^{3}X,\ldots \right\} \end{array}$$

we define the instrument matrix^1^

$$\begin{array}{l} Z=\left\{X,WX\right\} \end{array}$$

Thus, generating the moment conditions via the identity:

$$\begin{array}{l} E\left[Z^{\prime }u\right]=0 \end{array}$$

$\hat{\theta }$ can be estimated by the following

(8) θ^=arg min Θu′ZΩZ′u

The estimation algorithm used in our application is explained in detail in section 2.3.

### 2.2. The Network

The spatial regression model we propose is based on an exogenously defined network, where the nodes correspond to individual companies and the ties express the volume of trade between any pair of companies, i.e., the trade flow from company *i* to company *j*, for each *i* and each *j*. This information is generally not available, so we must approximate it using data on aggregate input-output trade between sectors.

The World Input Output Trade (WIOT) statistics provide information on the aggregate trade volumes of 52 economic sectors in each country with all sectors in all countries.

For a given country, define *A* as the sector of company *i*, *B* as the sector of company *j*, and let *f*_*AB*_ be the trade flow from sector A to sector B, while *f*_*BA*_ is the trade flow from sector B to sector A.

Replacing the individual flows with the aggregate ones, the entries of the approximate trade matrix *F* are then obtained as:

$$\begin{array}{l} f_{ij}=f_{AB}=\underset{l\in A}{\sum }\underset{m\in B}{\sum }f_{lm} \end{array}$$

To use these data for proxying the individual companies' flows, we need to calculate the proportion of each company in terms of size over its sector using a suitable measure, such as turnover or the value of trade receivables (for inflows) and payables (for outflows).

Consider, for example, the case of determining the trade flows from company *i*, belonging to sector *A*, to company *j*, belonging to sector *B*, knowing the individual trade payables and receivables.

We first calculate the ratio between company *i* trade payables $\tilde{x_{i}}$ and the sum of sector *A* trade payables:

$$\begin{array}{l} x_{i}=\frac{{\tilde{x}}_{i}}{\sum_{l\in A}{\tilde{x}}_{l}} \end{array}$$

Then we calculate the ratio between company *j* trade receivables $\tilde{y_{i}}$ and the sum of sector *B* trade receivables:

$$\begin{array}{l} y_{j}=\frac{{ỹ}_{j}}{\sum_{m\in B}{ỹ}_{m}} \end{array}$$

The product *x*_*i*_*y*_*j*_ is a proxy of the proportion of flows from company *i* to company *j* on the total flows from sector *A* to sector *B*.

Repeating this calculation for all companies, we get the matrix:

$$\begin{array}{l} R=\langle x,y\rangle =\left(\begin{array}{cccc} x_{1}y_{1} & x_{1}y_{2} & \cdots & x_{1}y_{n}\\ x_{2}y_{1} & x_{2}y_{2} & \cdots & x_{2}y_{n}\\ \vdots & \ddots & \cdots & \vdots \\ x_{n}y_{1} & x_{n}y_{2} & \cdots & x_{n}y_{n} \end{array}\right) \end{array}$$

Finally, by calculating the entrywise product of *R* and the trade matrix *F*, we get the following matrix:

$$\begin{array}{l} W=R\circ F=\left(\begin{array}{cccc} x_{1}y_{1}F_{1,1} & x_{1}y_{2}F_{1,2} & \cdots & x_{1}y_{n}F_{1,n}\\ x_{2}y_{1}F_{2,1} & x_{2}y_{2}F_{2,2} & \cdots & x_{2}y_{n}F_{2,n}\\ \vdots & \ddots & \cdots & \vdots \\ x_{n}y_{1}F_{n,1} & x_{n}y_{2}F_{n,2} & \cdots & x_{n}y_{n}F_{n,n} \end{array}\right) \end{array}$$

Note that the *ij* element can be interpreted as the proxy of the trade flow from company *i* to company *j*. Conversely, the *ji* element can be interpreted as the proxy of the trade flow from company *j* to company *i*. The estimated flows define the magnitude of intercompany connections. To use *W* as a spatial weighting matrix in our application, we need to set the entries on the diagonal to 0 and normalize the rows so as to sum to 1.

### 2.3. Estimation Procedure

To estimate the SAR model parameters, we use a two-step estimation procedure:

- minimize Equation (8), letting Ω = *I*_2*k*−1_, to obtain parameter estimates $\hat{\theta }$ and calculate the optimal weighting matrix by computing the covariance of the moments:
  $$\begin{array}{l} Ŝ=\frac{1}{n}Z^{\prime }uu^{\prime }Z \end{array}$$
  where the residual vector *u* is calculated as in (7).
- recompute the parameter estimates $\hat{\theta }$ by substituting the identity matrix with the optimal weight matrix:
  $$\begin{array}{l} \hat{\Omega }={Ŝ}^{-1} \end{array}$$
  Note that this procedure requires inversion and multiplication of large matrices, so the computation time can be very long when working with large datasets. Possible solutions should be based on suitable simplifications to the connectivity matrix *W* to make it more sparse, such as fixing a threshold for the relevance of trade flows. However, with our sample size (*n* = 1, 185) the computational time for the two-step algorithm is more than acceptable. We remark that the employed data is available as Supplementary Material.

## 3. Data and Results

In this section we empirically verify whether the predictive performance of P2P credit scoring models can be improved using correlation network models. In particular, we are interested in assessing significance and magnitude of the contagion parameter ρ. The more the contagion parameter is close to 1, the more the networking information can support credit risk evaluation. To achieve this goal, we have collected data from a European Credit Assessment Institution (ECAI), that supplies credit scorings to P2P platforms specialized in business lending. We use data relative to 1,185 borrowing Italian SMEs, in 2015–2016. The proportion of observed defaults in our sample is nearly 11%, which is large, in line with the observed impact of the recent financial crisis in Southern European countries. The available data include the status of the companies, classified as [1 = Defaulted] and [0 = Active], in 2016 as well as some main financial information, for year 2015. From the available data, we select three financial ratios reflecting the three most important aspects related to default probability: operational performance, business sustainability and financial sustainability. Specifically, we consider:

- the return on equity ratio (RATIO012)
- the activity ratio, expressed as the ratio between sales and total assets (RATIO018);
- the solvency ratio, expressed as the ratio between the net income and the total debt (RATIO027)

**Figure 1:**
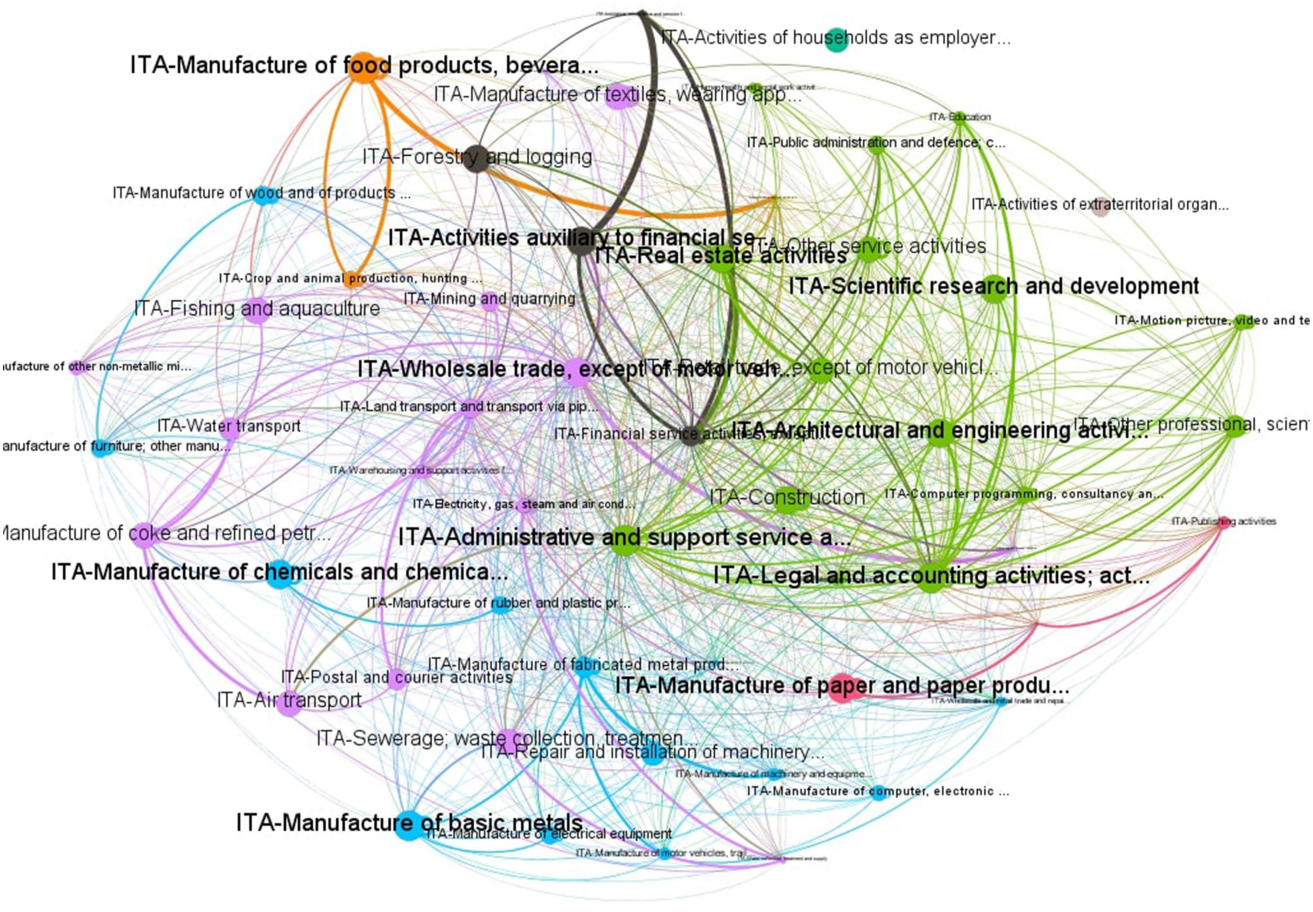
Network of P2P Italian SMEs.

The spatial weight matrix *W* has been built from the WIOT database, as described in section 2.2 and using turnover as a company size measure. Figure 1 shows the network based on the estimated connections.

Table 1 shows the parameter estimates obtained using a simple logit model, without the spatial component.

**Table 1:** Results of estimation of non-spatial logit model.

	**Estimate**	**Std. error**	**Pr(> |*z*|)**
Intercept	−2.11	0.16	2.97e–38
β_1_ (RATIO012)	−0.69	0.10	6.35e–11
β_2_ (RATIO018)	0.02	0.10	0.84
β_3_ (RATIO027)	−0.01	0.00	9.10e–04

Then we estimate the SAR model (3) through the algorithm presented in section 2.3. The obtained results are reported in Table 2.

**Table 2:** Results of estimation of SAR model.

	**Estimate**	**Std. error**	**Pr(> |*z*|)**
ρ	0.78	0.23	5.44e–04
Intercept	0.44	0.46	0.35
β_1_ (RATIO012)	−0.53	0.15	2.24e–04
β_2_ (RATIO018)	0.05	0.13	0.69
β_3_ (RATIO027)	−0.03	0.01	0.03

We first note from Table 2 that the contagion parameter is significant and its value is high (0.78). The effect of financial ratios is stable, supporting the SAR specification including both a spatial and an exogenous component. Thus, considering a measure of connectivity between companies significantly explains the credit risk arising from P2P lending, improving the traditional analysis based on individual financial indicators.

**Figure 2:**
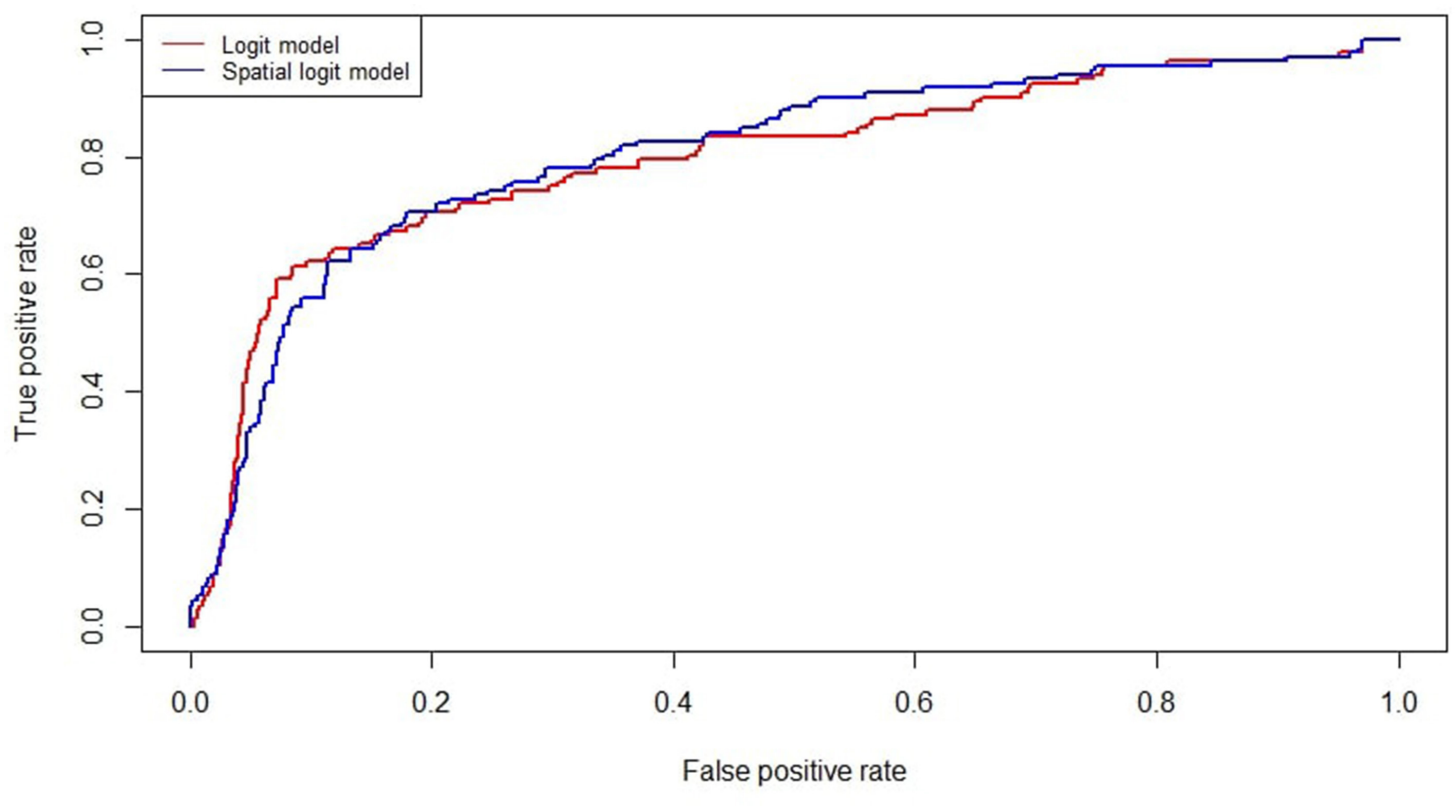
ROC curves of baseline and spatial logit models.

Including the spatial component also improves model accuracy, as shown in Figure 2 plotting the ROC curves of the simple logit and the spatial logit model. The AUC (Area Under the ROC Curve) values are 0.798 and 0.806, respectively. It is worth noting that the difference in the AUC values is modest and could turn out to be non-significant in an out-of-sample exercise. However, the proposed specification defines a contagion model which can support the analysis of interconnectedness between the agents' default risk, even when this does not improve the predictive performance in a crucial way. Future research may concern dealing with unbalanced samples (as in Calabrese and Giudici, 2015) and/or with multiple data sources (as in Figini and Giudici, 2011).

## 4. Conclusions

This paper provides a method, based on binary spatial regression models, to improve default prediction by estimating the interdependence between companies due to trade ties.

We have applied the methodology to a sample of Italian companies, finding evidence of a high level of spatial autocorrelation, interpretable as a credit contagion parameter.

The proposed model provides both a description of contagion (through the spatial component) and a predictive capability, differently from most existing contagion models, which provide either of the two. The model can be easily implemented, as a modification of a classical logistic regression that includes interconnectedness. We believe that the findings which can be derived from spatial autoregressive models may be useful, especially for P2P lenders who can use it to improve credit risk assessment.

From a methodological viewpoint, further research may involve employing a different generalized linear model, such as the generalized extreme value regression models discussed in Calabrese and Elkink (2016). Moreover, the dependence structure could be extended to the dynamic case (Arakelian and Dellaportas, 2012).

## Data Availability

The datasets for this manuscript are not publicly available because the data were provided by a private company. Requests to access the datasets should be directed to paolo.giudici@unipv.it.

## Author Contributions

All authors listed have made a substantial, direct and intellectual contribution to the work, and approved it for publication.

### Conflict of Interest Statement

The authors declare that the research was conducted in the absence of any commercial or financial relationships that could be construed as a potential conflict of interest.

## Footnotes

^1^As explained in Kelijian and Prucha (2010), *H* proxies the expected value of *WY*^*^ using its projection on *X*.
